# Cellulase-Assisted Extraction of Polysaccharides from White Hyacinth Bean: Characterization of Antioxidant Activity and Promotion for Probiotics Proliferation

**DOI:** 10.3390/molecules22101764

**Published:** 2017-10-20

**Authors:** Guo-Wei Shu, Yun-Xia He, Ni Lei, Ji-Li Cao, He Chen, Li Chen

**Affiliations:** 1 School of Food and Biological Engineering, Shaanxi University of Science and Technology, Xi’an 710021, China; shuguowei@gmail.com (G.-W.S.); lily46569@163.com(N.L.); chenhe419@gmail.com (H.C.); 2 Department of Research and Development, Xi’an Oriental Dairy Co., Ltd., Xi’an 710027, China; xiandfcjl@gmail.com; 3 College of Food Engineering and Nutritional Science, Shaanxi Normal University, Xi’an 710119, China

**Keywords:** white hyacinth bean polysaccharides, cellulase-assisted extraction, probiotics proliferation, antioxidant activities, response surface methodology

## Abstract

Food-derived polysaccharides have advantages over synthetical compounds and have attracted interest globally for decades. In this study, we optimized the cellulase-assisted extraction of polysaccharides from white hyacinth bean (PWBs) with the aid of response surface methodology (RSM). The optimum extraction parameters were a pH of 7.79, a cellulase of 2.73%, and a ratio of water to material of 61.39, producing a high polysaccharide yield (3.32 ± 0.03)%. The scavenging ability of PWBs varied on three radicals (hydroxyl > 2,2-diphenyl-1-picrylhydrazyl (DPPH) > superoxide). Furthermore, PWBs contributed to the proliferation of three probiotic bacteria (*Lactobacillus acidophilus* LA5, *Bifidobacterium bifidum* BB01, and *Lactobacillus bulgaricus* LB6). These investigations of PWBs provide a novel bioresource for the exploitation of antioxidant and probiotic bacterial proliferation.

## 1. Introduction

Reactive oxygen species (ROS) is the chemical active products produced by the partial reduction of oxygen [1], which contributes to various pathological processes such as aging, inflammation, cancer, and atherosclerosis [2,3]. Free radicals can severely impact the quality and safety of food products due to lipid peroxidation [4]. Scavenging free radicals could weaken oxidative activity, which brings on a higher nutritional quality and a longer shelf-life of product. Therefore, the application of synthetic antioxidants in food and cosmetics has been well developed [5]. However, it has been reported that synthetic antioxidant might be related to neoplasia and liver damage [6]. Natural antioxidants such as polysaccharides from plants are characterized by excellent antioxidant activity and nontoxicity. Petera et al. [7] reported that polysaccharides extracted from *Cereus triangularis* cladodes showed lower antioxidants activities than mucilage extracted from peeled *Opuntia ficus-indica* fruits. Polysaccharide from *Phellinus nigricans* mycelia exhibited positive radical-scavenging activities against superoxide anion, hydroxyl, and 2,2-diphenyl-1-picrylhydrazyl (DPPH) radicals [8]. Delattre et al. [9] found that the modification of polysaccharides via regioselective oxidation had a positive effect on scavenging DPPH radical ability. Replacing synthetic antioxidants with natural substances has been a focus of pharmacologists and biologists for decades [10,11,12,13].

White hyacinth bean, the mature seed of *Dolichos lablab* L., has the concomitant function of both medicine and foodstuff. It has been widely planted in tropic and subtropic areas [14]. Polysaccharides are vital components of white hyacinth bean and are highly correlated with antioxidant activity. Common methods of extracting natural polysaccharides include ultrasound-assisted extraction, microwave-assisted extraction, acidic hydrolysis, and enzyme-assisted extraction. Enzyme-assisted extraction possesses several advantages, such as high efficient and extraction yield, low energy consumption, high efficiency, and easy operation [15]. However, the enzyme-assisted extraction of polysaccharides from white hyacinth bean (PWBs) and the application of these polysaccharides as antioxidants are not common due to a scarcity of the requisite materials.

Probiotic bacteria are living microorganisms that, when administered in adequate amounts, confer a health benefit on the host, and they are essential to the homeostasis and health of the gastrointestinal tract for human beings. Prebiotics are the non-digestible food ingredients that are actually used to stimulate the growth of probiotics. The combination of probiotics and prebiotics has a synergic effect on host health. The extraction of polysaccharides from natural sources is currently gaining attention for their potential prebiotic properties. Polysaccharides served as prebiotics could advance the growth of beneficial lactic acid bacteria in the colon [16].

We have carried out single-factor tests to study the effects of cellulase, pectinase, and xylanase on the extraction of PWBs. The Plackett-Burman and steepest ascent experiment showed that pH, the addition amount of cellulase and the ratio of water to material were main factors for polysaccharide yield [17]. In the present study, the extraction conditions of PWBs were optimized by a central composite design (CCD). The scavenging activity of PWBs with respect to DPPH radicals, hydroxyl radicals, and superoxide radicals was determined. To our knowledge, studies concerning the prebiotic activity of PWBs are rare. Thus, the effect of PWBs on the growth of three selected probiotics—*Lactobacillus bulgaricus* (LB6), *Lactobacillus acidophilus* (LA5), and *Bifidobacterium bifidum* (BB01)—was also studied.

## 2. Results and Discussion

### 2.1. Optimization of Cellulase-Assisted Extraction of PWBs by Response Surface Methodology

The optimal combination of three independent variables, pH value (A), cellulase (B), and the ratio of water to material (C), was studied using a CCD. The experimental design and the result of response value Y (polysaccharide yield) according to RSM are given in Table 1.

Design-Expert software was used to build the response surface model and analyze the data given in Table 2. The regression equation of this experiment is

(1) R1=3.31+0.10A+0.10B−0.16C−0.07AB+0.14AC−0.21BC−0.17A2−0.35B2−0.16C2

In this equation, R1 means the polysaccharide yield. A, B, and C represent the coded values of pH value, cellulase (%), and the ratio of water to material, respectively.

The regression equation obtained was evaluated by analysis of variance (ANOVA) and tested the level of significance. The results are shown in Table 2. The *p*-value of the model obtained was smaller than 0.001, showing the high significance of the regression model. In addition, all of the one-degree term and squares of this regression equation are significant, which suggests the relation between the response value and the factors are not a simple linear relation. The *p*-value of AC was lower than 0.01, which represents a significant interaction between pH and the ratio of water to material. A similar interaction was observed between this ratio and cellulase. However, the interaction between pH and cellulase was non-significant. The coefficient of the R^2^ value of 0.9620 illustrated that 96.20% of the total variation in the response was explained by the model. The AdjR^2^ value of 0.9278 close to the R^2^ value showed the significance of the model, which indicated a good relationship between the predictive and the measured value of PWB yield. The value of PRESS (predicted residual sum of square) was 0.996, which indicates that the degree of fitting is workable. The SNR (signal-to-noise ratio) of 4.081 (>4) implied that the model is satisfactory. Adequate precision is a measure of the range in predicted response relative to its associated error. The adequate precision value was 14.2808 greater than 4, indicating that the models of response surface can be used to navigate the design space. Thus, it can be concluded that the model is adequate to describe the PWB extraction yield via RSM.

**Figure 1:**
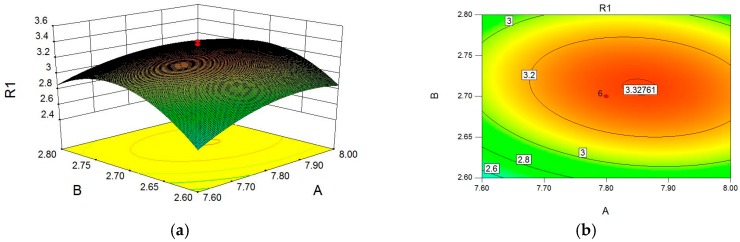

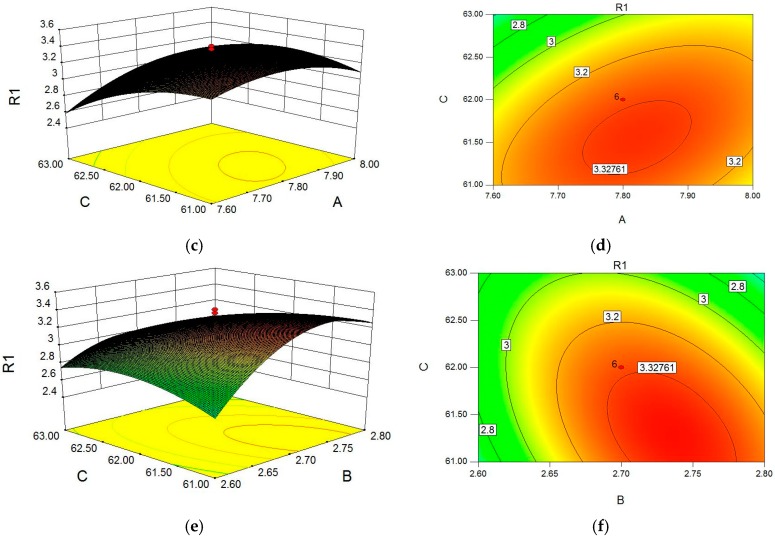
Response surface plot and contour plot showing interaction effects of A (pH value) and B (cellulase) (**a**,**b**), A (pH value) and C (ratio of water to material) (**c**,**d**), B (cellulase) and C (ratio of water to material (**e**,**f**) on the polysaccharide yield.

Using software (Design-Expert 8.0.6) to analyze the regression equation, the response surface plot and contour plot obtained with Equation (1) were produced and are shown in Figure 1.

**Figure 2:**
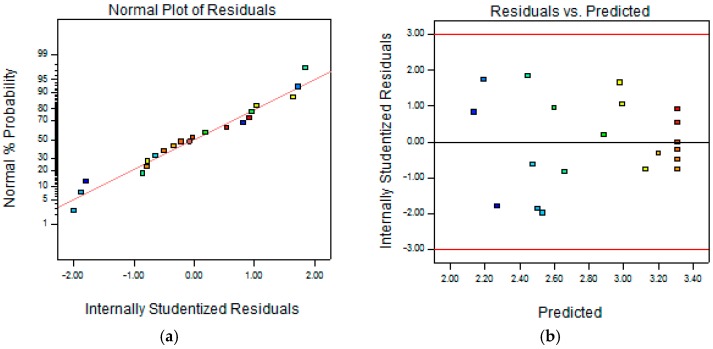
(**a**) Normal probability of internally studentized residuals; (**b**) Plot of internally studentized residuals vs. predicted response.

It is necessary to check the fitted model to ensure that it provides an adequate approximation to the real system. The investigation and optimization of the fitted response surface likely give misleading results if the model is not very adequate. The residuals in the least squares fit are very vital in checking the adequacy of model. By constructing a normal probability plot of the residuals, a check was made for the normality assumption, as given in Figure 2. The normality assumption was satisfied as the residual plot approximated along a straight line. Figure 2 also presents a plot of residuals vs. the predicted response. The general impression is that the residuals scatter randomly on the display, suggesting that the variance of the original observation is constant for all values of Y. These two plots are satisfactory.

By solving the inverse matrix of the quadratic polynomial equation employing the software of Design-Expert 8.0.6, the optimum values of the tested independent variables were a pH of 7.79, a cellulase of 2.73%, and a ratio of water to material of 61.39. Under the optimum conditions, the predicted polysaccharide yield reached a maximum of 3.37%. The confirmatory experiments were conducted under optimized conditions in order to test and verify the optimized result. Under the determined conditions, the mean value of polysaccharide yield was (3.32 ± 0.03)%, which is slightly lower than the predicted maximum value, indicating the model designed in this study is valid to optimize the extraction of polysaccharide.

### 2.2. Antioxidant Activities of PWBs

#### 2.2.1. Hydroxyl Radical-Scavenging Activity

**Figure 3:**
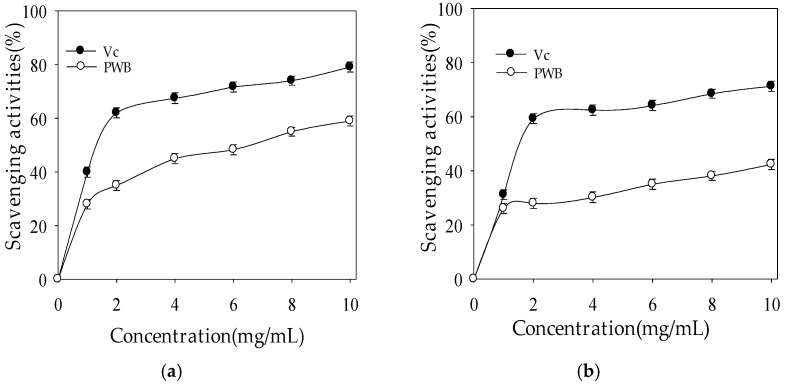

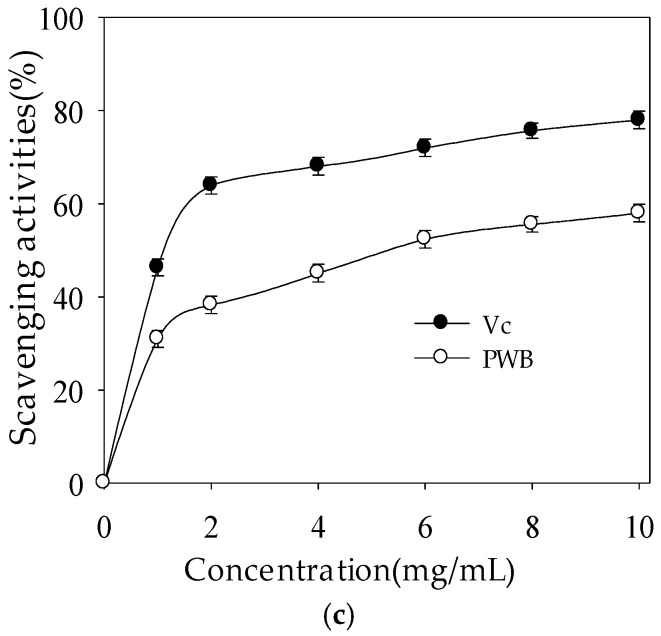
Scavenging activities of PWBs and ascorbic acid (V_C_) with respect to the hydroxyl radicals (**a**); superoxide radicals (**b**); and DPPH radicals (**c**). All values are represented as mean ± standard deviation (*n* = 3).

Hydroxyl radicals have been identified as strong oxidants that can produce severe damage to the biomolecules [18], so it is necessary to scavenge or decrease the concentration of hydroxyl radicals. The scavenging hydroxyl radical activities of PWBs and ascorbic acid (V_C_) are shown in Figure 3a. It is clear that the PWBs and V_C_ both had the ability to scavenge hydroxyl radicals. As shown in Figure 3a, the scavenging ability varied in a concentration-dependent manner, which grew with the increase in the concentration of PWBs and V_C_. The scavenging activity against hydroxyl radicals showed rapid growth at the low concentration of 2 mg·mL^−1^, which was 35% and 62.05% for PWBs and ascorbic acid, respectively. The scavenging activity of PWBs was lower than that of Vc within the tested concentration range. When the PWB concentration increased to 10 mg·mL^−1^, the scavenging activity reached 59%, which was equal to the 79.12% of V_C_ (used as standard) scavenging activity at same concentration. These results suggested that PWBs had significant scavenging ability with respect to hydroxyl radicals. The mechanism of the hydroxyl radical-scavenging activity of PWBs might be that the existence of numerous hydrogen donors in the polyhydroxy structures of PWBs, which have a strong affinity for hydroxyl radicals, can quickly capture hydrogen atoms in C-H chains and produce water. The carbon atom decomposes into harmless products after a range of reactions, resulting in the termination of free radical chain reactions [19].

#### 2.2.2. Superoxide Radical-Scavenging Activity

Superoxide anion radicals, some of the strongest reactive oxygen species among the free radicals, can be converted to harmful ROS such as hydrogen peroxide, hydroxyl radicals, and damaging biomolecules that result in chronic diseases [20]. Superoxide anion radicals are generated by enzyme systems, such as peroxidase, NADPH oxidase, and xanthine oxidase. Antioxidants can delay the oxidation process by inhibiting the polymerization chain initiated by free radicals and other subsequent oxidizing reactions. When superoxide anion radicals were scavenged by antioxidants, the absorbance at 320 nm increased due to the color change from purple to yellow [21], which indicates the antioxidant ability of polysaccharides. The results of scavenging the superoxide radical activities of polysaccharides and ascorbic acid can be seen in Figure 3b. Similar to the scavenging hydroxyl radical activities, the scavenging activities of polysaccharides and ascorbic acid on superoxide radicals increased with the rise in concentration. At the high concentration of 10 mg·mL^−1^, the levels of superoxide radical scavenging were 42.4% and 71.3% for PWBs and ascorbic acid, respectively. The mechanism of superoxide radical-scavenging activity might be that an oxidation reaction occurs between PWBs and superoxide anion radicals, contributing to the effect of scavenging superoxide radicals [19].

#### 2.2.3. DPPH Radical-Scavenging Activity

DPPH radicals, important compounds that possess proton free radicals, decrease greatly when exposed to proton radical scavengers [22]. Figure 3c depicts the scavenging ability of polysaccharides and ascorbic acid with respect to DPPH radicals. The results suggest that polysaccharides and ascorbic acid can both scavenge DPPH radicals. There was a positive correlation between scavenging activity and polysaccharide concentration. However, it was noted that, for all concentrations (0 to 10 mg·mL^−1^), the DPPH radical scavenging activities of polysaccharides were always lower than those of ascorbic acid. When polysaccharides amounted to 10 mg·mL^−1^, the scavenging activity with respect to DPPH radicals reached 58%, which was almost 78% of the scavenging activity of V_C_ at the same concentration. The scavenging activity of PWBs in terms of DPPH may be due to the existence of the numerous hydroxyls in the polysaccharide molecule, which might serve as an electron donator and transfer electrons to DPPH free radicals [23]. Therefore, polysaccharides and ascorbic acid both showed scavenging activity on the hydroxyl radicals, superoxide radicals, and DPPH radicals. However, their scavenging abilities were different. These observations suggest that the scavenging activity of polysaccharides was lower compared to that of the V_C_. It has been proven that polysaccharides obtained from plants have excellent antioxidant activity. Previous research has shown that polysaccharides contribute greatly to anti-tumor activity, lower blood pressure, and cholesterol [11]. The antioxidant activity of polysaccharides could be affected by many different factors, such as their chemical structure, molecular weight, degree of branching, and chemical composition [24,25]. PWBs are crude and water-soluble, so it is necessary and essential to complete further purification.

### 2.3. Effect of Polysaccharide on the Growth of Three Selected Probiotics

**Figure 4:**
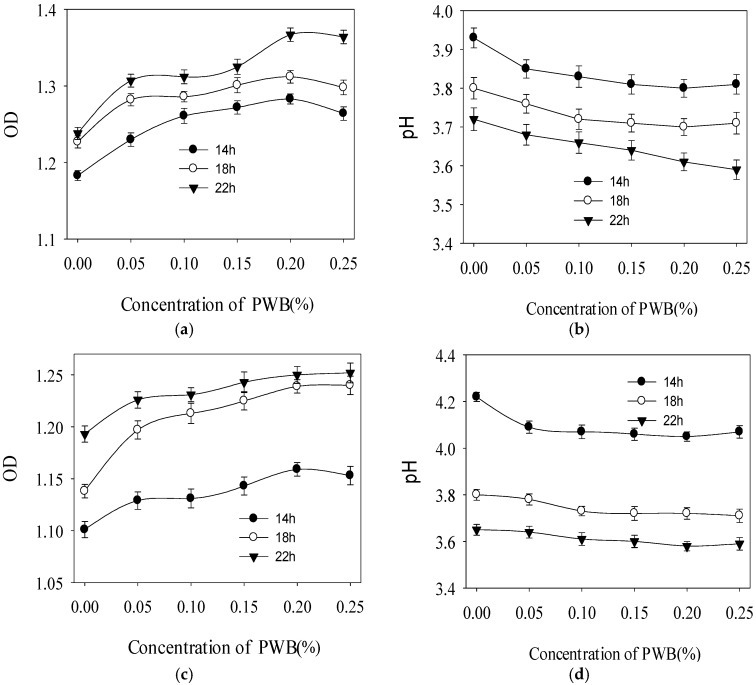

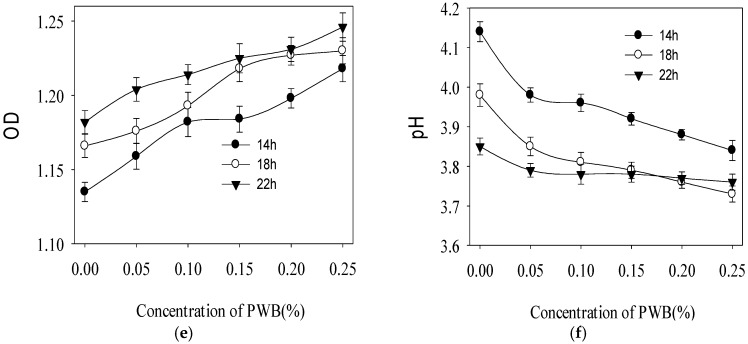
Effects of PWBs on the growth of *Lactobacillus acidophilus* LA5 (**a**,**b**), *Bifidobacterium bifidum* BB01 (**c**,**d**), and *Lactobacillus bulgaricus* LB6 (**e**,**f**). All values are represented as mean ± standard deviation (*n* = 3).

The effect of polysaccharides on the growth of *L. acidophilus* LA5 is shown in Figure 4a,b. It suggested that the promotion effect of polysaccharides on *L. acidophilus* LA5 was related to the addition of polysaccharide. Within 0.2%, the promotion effect of polysaccharides on *L. acidophilus* LA5 increased as the addition of polysaccharide. When the polysaccharide addition was 0.20%, the OD value and pH value tended to be stable. These could be explained by the change of osmotic pressure and pH value. Metabolites accumulated and finally resulted in limitation of *L. acidophilus* LA5 proliferation. The effect of polysaccharides on the growth of *B. bifidum* BB01 was shown in Figure 4c,d. Similar to the *L. acidophilus* LA5, a concentration-dependent manner was observed for the prebiotic effect of PWBs on the growth of *B. bifidum* BB01. When the addition was 0.20%, the OD value and the pH value were tended to be stable. Figure 4e,f showed the effect of polysaccharides on the growth of *L. bulgaricus* LB6. The results suggested that the promotion effect of polysaccharides on *L. bulgaricus* LB6 was related to the addition of polysaccharide. The promotion effect had an obvious enhancement with the increase of polysaccharide addition.

Taken together, PWBs exhibited a different prebiotic effect on these three probiotics (*L. acidophilus* LA5, *B. bifidum* BB01, and *L. bulgaricus* LB6). The ability of probiotics to degrade and ferment polysaccharides varies greatly among different species of LAB even among strains of the same species [26]. *L. acidophilus* LA5, *B. bifidum* BB01, and *L. bulgaricus* LB6 are three common probiotics, which have been used in many foods, especially yogurt. Similar results were found by Mueller [27], who found that the neutral polysaccharides from *Hyptis suaveolens* significantly induced the growth of *L. acidophilus*, *L. paracasei* DN114001, DSM20312, and CRL431, *L. reuteri*, *L. rhamnosus GG*, *B. infantis*, *L. brevis*, *Lc. lactis*, *L. fermentum*, and *S. thermophilus*. Madhukumar and Muralikrishna [28] reported that xylooligosaccharides extracted from bengal gram husk and wheat bran served as active prebiotic components. Numerous studies on the prebiotic potential of polysaccharides extracted from natural sources have been reported such as brown seaweed [29] and ginseng [30]. Polysaccharides served as a carbon resource in the metabolic process of probiotics can be disintegrated into monosaccharides, which can provide energy for probiotics. Meanwhile, probiotics can produce organic acid in the utilization process of polysaccharides, which contribute greatly to the growth of probiotics. The prebiotic effect of polysaccharides could be affected by its chemical structure, its degree of branching, its solubility in water, and its degree of polymerization, among other factors [31].

PWBs have a positive effect on three probiotics, which indicates that PWBs encourage the proliferation of probiotics. Oligosaccharides are prebiotics that are widely used in yogurt. Prebiotics can be hydrolyzed by the enzymes in probiotics. Therefore, probiotics can hydrolyze and utilize carbohydrate prebiotics to produce energy and promote growth of probiotics.

## 3. Materials and Methods

### 3.1. Materials and Chemicals

White hyacinth beans (Batch number: 160101) were purchased from Xi’an Zaolutang Pharmaceutical Co., Ltd. (Xi’an, China). The samples were cleaned and dried before extraction. The bacteria used in this study were *Lactobacillus bulgaricus* LB6, *Lactobacillus acidophilus* LA5, and *Bifidobacterium bifidum* BB01, which were obtained from the School of Food and Biological Engineering, Shaanxi University of Science and Technology. Cellulase (10,000 U·g^−1^) was purchased from Zhaodong Beifang Enzyme Prepartion Co., Ltd. (Zhaodong, Heilongjiang, China). 2,2-Diphenyl-1-picrylhydrazy radicals (DPPH) were purchased from Yobios Co., Ltd. (Xi’an, China). All other chemicals were of analytical grade.

### 3.2. Enzyme-Assisted Extraction of PWBs

Dried white hyacinth beans were pulverized and sifted through a 60 mesh sieve to obtain a homogeneous powder. The dried powder was dispersed in distilled water (water to material ratio, 60.32–63.68 mL·g^−1^). The pH of the suspension was adjusted to a designated value (7.46–8.14), and cellulase (2.53–2.87%) was added. The reaction system was placed in a water bath at 60 °C for 3 h. After the enzymolysis process, samples were rapidly heated at 90 °C for 10 min. The extract solution was cooled, filtered, and concentrated. After centrifugation at 4000 rpm for 10 min, the crude extract was mixed with 3 volumes of ethanol and stored at 4 °C overnight. The protein in the crude polysaccharides was removed via the Sevag method [32]. Afterwards, the 95% (*v·v*^−1^) ethanol was added to precipitate the polysaccharide, filtered via filter paper under suction funnel, dissolved with distilled water, and centrifuged (4000 rpm for 10 min). Finally, the supernatant was suitably diluted and the content of PWBs in the extract was evaluated via the phenol-sulfuric method using glucose as a standard [33]. The yield of PWBs was calculated by the following equations:

(2) Polysaccharideyield(%)=polysaccharideweight(g)whitehyacinthbeanweight(g)×100

### 3.3. Central Composite Design (CCD) and Statistical Analysis

According to the results of single factor test and Plackett-Burman experiment, a central composite design was employed to optimize the extraction conditions. Each group conducted three parallel experiments. The coded values of variables were determined according to the following equation:

(3) $$x_{i}\;=\;\frac{X_{i}-X_{z}}{{\Delta X}_{i}},\;i\;=\;1,2,3........k$$

where x_i_ is the dimensionless coded value of an independent variable; X_i_ is the true value of an independent variable; X_z_ represents the true value of an independent variable at the center point; ∆X_i_ is step change of the real value of the variable i [34]. The coded values of different levels of each variable are shown in Table 3.

A quadratic polynomial mathematical equation based on the data obtained from CCD was utilized to express the relationships between the variables. The developed equation in terms of the coded process variables are given below:

(4) $$R1\;=\;\beta_{0}+\beta_{1}A+\beta_{2}B+\beta_{3}C+\beta_{12}\mathrm{AB}+\beta_{13}\mathrm{AC}+\beta_{23}\mathrm{BC}+\beta_{11}A^{2}+\beta_{22}B^{2}+\beta_{33}C^{2}$$

where R1 is the response value (polysaccharide yield), and A, B, and C represent the coded values of the pH value, the cellulase, and the ratio of water to material, respectively. β_0_ is the constant regression coefficient. (β_1_, β_2_, β_3_), (β_11_, β_22_, β_33_), and (β_12_, β_23_, β_13_) are the regression coefficient for the linear, quadratic, and interaction terms, respectively.

Design Expert 8.0.6 software (Stat-Ease. Inc., Minneapolis, MN, USA) was utilized to investigate the experimental data statistically. Multiple regression analysis was applied to experimental data to construct a quadratic polynomial mathematical model for the response.

### 3.4. Antioxidant Activity of Polysaccharides

#### 3.4.1. An Assay of Scavenging Hydroxyl Radical Activity

The scavenging ability of PWBs on hydroxyl radicals was measured according to the Fenton method [35] with some modifications. The reaction mixture contained 2 mL of a phosphate buffer (0.2 mol·L^−1^, pH 7.4), 2 mL of an orthophenanthroline solution (0.1 mmol·L^−1^), 1 mL of a ferrous sulfate (FeSO_4_) solution (0.15 mmol·L^−1^), 1 mL of a hydrogen peroxide (H_2_O_2_) solution (0.01%), and 1 mL of samples. After incubation at 37 °C for 60 min, the absorbance of different mixtures was measured at 510 nm with a spectrophotometer. Ascorbic acid (V_C_) was used as a reference material, and all tests were carried out in triplicate. The scavenging activity of polysaccharides on hydroxyl radicals was calculated using the following equation:

(5) Scavengingactivity(%)=[(A2−A0)/(A1−A0)]×100

where A_0_ is the absorbance of the reaction solution without PWBs, A_2_ is the absorbance of the sample with the H_2_O_2_ solution, and A_1_ is the absorbance of the sample under conditions similar to that of A_2_, but with water instead of an H_2_O_2_ solution.

#### 3.4.2. Assay of Superoxide Radical Scavenging Activity

The activity of scavenging superoxide radicals was tested via the auto-oxidation of pyrogallic acid as in [36] but with a slight modification. In this experiment, the reaction mixture contained 2.5 mL of a Tris-HCl buffer (0.05 mol·L^−1^, pH 8.2), 0.1 mL of a pyrogallic acid solution (0.01 mol·L^−1^), and 0.4 mL of a polysaccharide solution. The polysaccharides and Tris-HCl were incubated at 25 °C for 20 min, and pyrogallic acid was then added to the mixture. After shaking, the absorbance of the sample at 325 nm was measured using a spectrophotometer. The scavenging activity of polysaccharides on superoxide radicals was calculated by the following equation:

(6) Scavengingactivity(%)=[(A0−A1)/A0]×100

where A_0_ is the absorbance of the control (without PWBs), and A_1_ is the absorbance of the mixture containing PWBs.

#### 3.4.3. Assay of DPPH Radical Scavenging Activity

The scavenging activity of polysaccharides on 2,2-diphenyl-1-picrylhydrazyl (DPPH) radicals was measured according to the method by Kazuko et al. [37] but with a slight modification. Two milliliters of an aliquot of a DPPH solution (0.2 mmol·L^−1^) in anhydrous ethanol and 2 mL of sample in different concentrations (1.0, 2.0, 4.0, 6.0, 8.0 and 10.0 mg·mL^−1^) were mixed together, and left to sit for 40 min after shaking. Ethanol with a concentration of 50% was used as the blank group. The scavenging ability was measured by determining the absorbance of the mixture at 517 nm, and all tests were carried out in triplicate. When proton radicals from DPPH are scavenged by polysaccharides, its purple color rapidly fades [38], which changes the absorption value at 517 nm. The scavenging activity of polysaccharides on DPPH radicals was calculated according to the following formula:

(7) Scavengingactivity(%)=[1−(Ai−Aj)/A0]×100

where A_0_ is the absorbance of the DPPH solution without the samples, A_i_ is the absorbance of the polysaccharides with the DPPH solution, and A_j_ is the absorbance of the sample under conditions similar to A_i_, but with water instead of the DPPH solution.

### 3.5. Effect of PWBs on the Growth of Three Selected Probiotics

An MRS medium (lactose: 20 g, peptone: 10 g, sodium acetate: 3 g, MgSO_4_: 0.2 g, MnSO_4_: 0.05 g, L-cysteine hydrochloride: 0.5 g, Tween 80: 1 mL, dissolved in 1000 mL of distilled water (pH of 6.2)) and PWBs were sterilized at 121 °C for 15 min. After cooling to room temperature, *L. acidophilus* LA5, *B. bifidum* BB01, and *L. bulgaricus* LB6 were incubated in the MRS medium (3% inoculum size) at 37 °C. Five different concentrations of PWBs (0.05%, 0.10%, 0.15%, 0.20%, and 0.25% in volume) were added to the medium. There were no polysaccharides in the blank group. The growth of three probiotics was assessed by determining the OD_600_ and the pH value of the culture solution every 4 h from the 14th hour (recorded 3 times in total).

## 4. Conclusions

In this study, cellulase-assisted extraction was optimized to extract PWBs. A CCD was employed to optimize the extraction process. The optimum parameters of PWB extraction obtained from the statistical analysis were as follows: a pH of 7.79, a cellulase of 2.73%, and a ratio of water to material of 61.39. Under these optimum conditions, the predicted polysaccharide yield reached a maximum of 3.37%. The confirmatory experiments were conducted to test and verify the optimized result. Under the determined conditions, the mean value of the polysaccharide yield was (3.32 ± 0.03)%. In addition, the PWBs showed different scavenging activity with respect to hydroxyl radicals, superoxide radicals, and DPPH radicals. The scavenging ability of PWBs with respect to these three radicals was lower than that of V_C_. Therefore, PWBs are potential natural antioxidants in functional food and medicinal products. Furthermore, our results indicate that PWBs might promote the growth of *L. acidophilus* LA5, *B. bifidum* BB01, and *L. bulgaricus* LB6 and can be employed as novel prebiotics.

## Figures and Tables

**Table 1:** The experimental design and results of the central composite design (CCD).

Run	Coded Variable Levels			Polysaccharide Yield Y (%)		
	A	B	C	Actual Value Y	Predicted Value Y’	Residual Y−Y’
1	1.682	0	0	3.07	2.99	0.076
2	−1	1	−1	3.18	3.20	−0.022
3	0	0	0	3.23	3.31	−0.082
4	−1.682	0	0	2.60	2.66	−0.062
5	−1	−1	1	2.15	2.27	−0.12
6	1	−1	−1	2.38	2.50	−0.12
7	0	0	0	3.41	3.31	0.098
8	0	0	−1.682	3.07	3.13	−0.056
9	−1	−1	−1	2.57	2.45	0.12
10	0	0	0	3.26	3.31	−0.052
11	1	1	−1	3.09	2.98	0.11
12	1	1	1	2.40	2.53	−0.13
13	0	0	0	3.37	3.31	0.058
14	0	0	0	3.31	3.21	−0.0021
15	0	1.682	0	2.43	2.48	−0.046
16	0	−1.682	0	2.20	2.14	0.060
17	0	0	1.682	2.67	2.60	0.069
18	0	0	0	3.29	3.31	−0.022
19	1	−1	1	2.90	2.89	0.013
20	−1	1	1	2.31	2.19	0.12

**Table 2:** The ANOVA of the CCD.

Source	SS	DF	MS	F	Pr > F	Sig
Model	3.409	9	0.379	28.109	<0.0001	***
A	0.134	1	0.134	9.909	0.0104	*
B	0.137	1	0.137	10.150	0.0097	**
C	0.333	1	0.333	24.714	0.0006	***
AB	0.039	1	0.039	2.909	0.1189	
AC	0.157	1	0.157	11.635	0.0066	**
BC	0.344	1	0.344	25.559	0.0005	***
A^2^	0.422	1	0.422	31.299	0.0002	***
B^2^	1.815	1	1.815	134.713	<0.0001	***
C^2^	0.363	1	0.363	26.935	0.0004	***
Residual error	0.135	10	0.013			
Lack of fit	0.112	5	0.022	4.889	0.0532	
Pure error	0.023	5	0.005			
Total	3.544	19				

DF: Degree of freedom; Pr: Probability; SS: Sum of squares; MS: Mean square; * *p* < 0.05, ** *p* < 0.01, *** *p* < 0.001, R^2^ = 96.20%, Radj^2^ = 92.78.

**Table 3:** The factors level coding table of the CCD.

Variable	Level				
	−1.682	−1	0	1	1.682
A, pH value	7.46	7.60	7.80	8.00	8.14
B, cellulase (%)	2.53	2.60	2.70	2.80	2.87
C, Ratio (*v*/*w*) of water (mL) to material (g)	60.32	61.00	62.00	63.00	63.68
